# A Large Inflammatory Myofibroblastic Tumor of the Urinary Bladder in a Parturient Treated by Partial Cystectomy: Case Report and Literature Review

**DOI:** 10.7759/cureus.29556

**Published:** 2022-09-25

**Authors:** Omar Buksh, Abdullah M Almalki, Ahmed Khogeer, Jaudah Al-Maghrabi, Mahmoud Alakraa

**Affiliations:** 1 Department of Urology, King Faisal Specialist Hospital and Research Centre, Jeddah, SAU; 2 Department of Pathology, Faculty of Medicine, King Abdulaziz University, Jeddah, SAU

**Keywords:** pseudotumor, myofibroblast, urinary bladder, anaplastic lymphoma kinase, inflammatory myofibroblastic tumor

## Abstract

Inflammatory myofibroblastic tumor (IMT) is a rare type of tumor composed mainly of fibroblastic and myofibroblastic spindle cells, with an inflammatory infiltrate of lymphocytes, plasma cells, and eosinophils. IMT may arise from different organs and sites, but it is infrequent to arise from the urinary bladder and usually manifests as hematuria. We report a case of a 24-year-old pregnant woman who presented to our hospital with gross hematuria. After further workup, we concluded that she had this extremely rare tumor, which was resected eventually with a partial cystectomy. Although the diagnosis of these kinds of tumors is usually made by anaplastic lymphoma kinase (ALK) using immunohistochemistry and detecting ALK gene translocation using fluorescence in situ hybridization (FISH), they were negative in our study; hence, we relied mainly on the morphological features of the tumor for the diagnosis.

## Introduction

Inflammatory myofibroblastic tumor (IMT) is a unique tumor that is classified by the World Health Organization (WHO) as an intermediate tumor (rarely metastasizing). This type of neoplasm comprises fibroblastic and myofibroblastic spindle cells, with an inflammatory infiltrate of lymphocytes, plasma cells, and eosinophils [1]. IMT may arise from different organs and sites, including the lungs, pelvis, and retroperitoneum. However, it infrequently arises from the urinary bladder [1]. When it develops in the urinary bladder, it usually manifests with lower urinary tract symptoms, hematuria, and bladder outlet obstruction [2]. We report a case of a 24-year-old pregnant woman who presented to our hospital with gross hematuria. After further workup, we concluded that she had this extremely rare tumor.

## Case presentation

**Figure 1:**
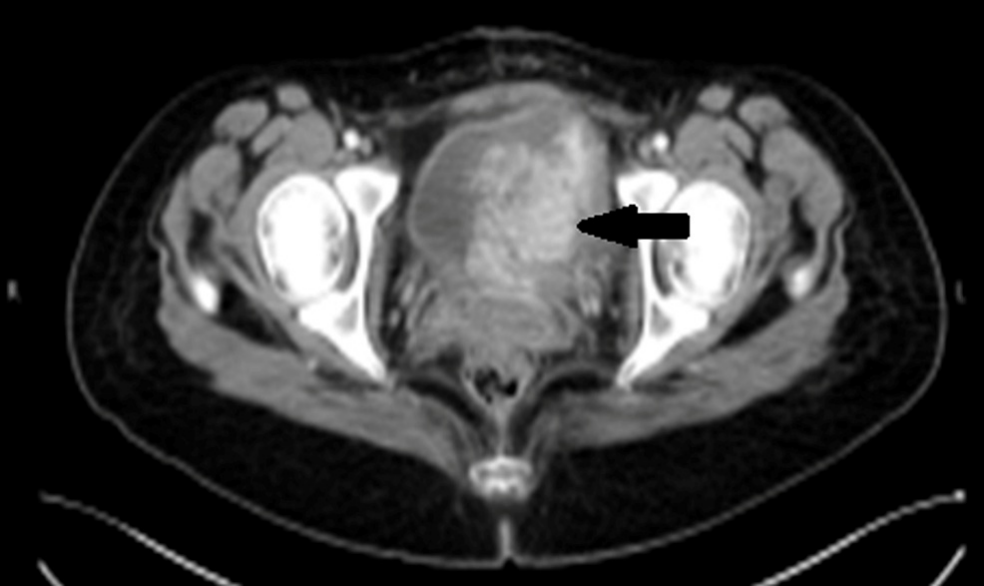
CT of the pelvis (axial section) showing large irregular soft tissue mass arising from the left lateral wall and base of the bladder, measuring 6.1 x 6.0 x 6.0 cm. CT, computed tomography

**Figure 2:**
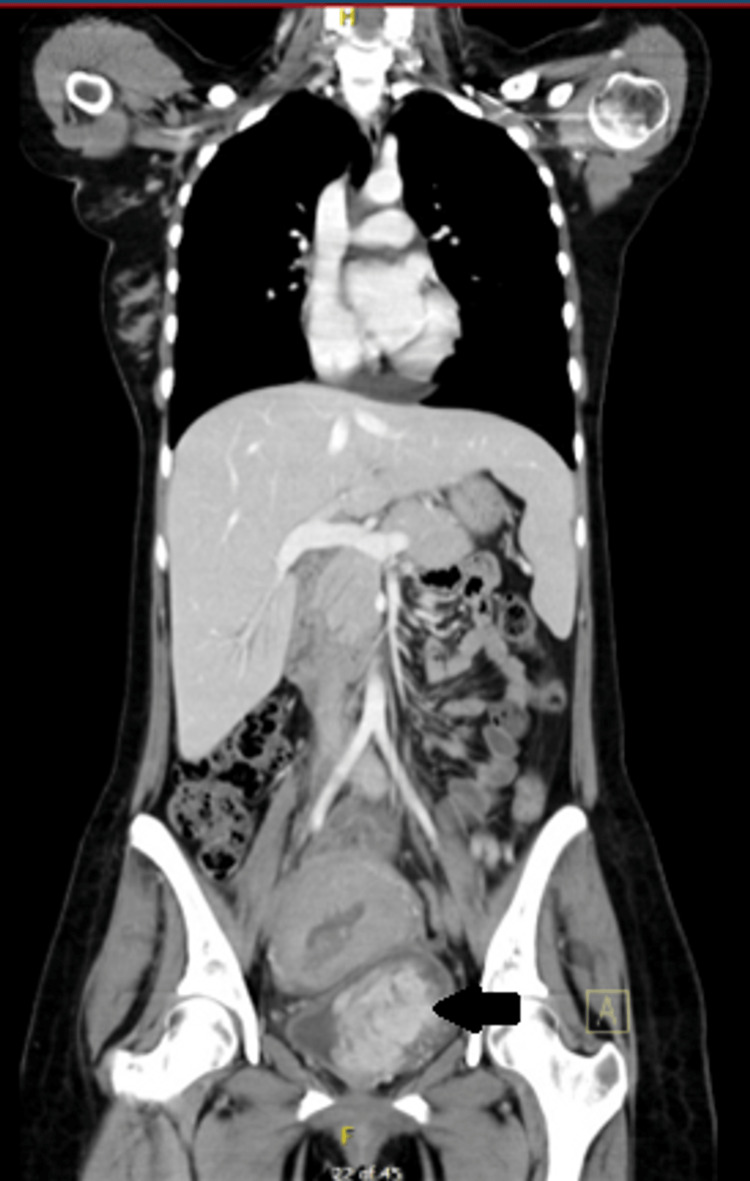
CT of the abdomen and pelvis (coronal section) showing a large irregular soft tissue mass arising from the left lateral wall and base of the bladder, measuring 6.1 x 6.0 x 6.0 cm, and encasing the right ureter at the ureterovesical junction. The uterus is anteverted and bulky, measuring 10.8 cm. CT, computed tomography

A 23-year-old female was referred to our hospital with a history of hematuria and clots for one month and increasing over the last three days before the current admission. Her hematuria was investigated by cystoscopy at another hospital during her 34th week of pregnancy, and a mass was identified, but no resection was performed at that time. Seven days prior to her admission to our hospital, the patient underwent elective cesarean section (CS) at her 36th week of pregnancy at another hospital due to the suspicious bladder mass and the possibility of it being malignant so that she can be referred sooner for tumor management. The decision to proceed with CS instead of spontaneous vaginal delivery (SVD) was made upon the patient’s preference, as she already had one CS previously. At the current presentation, she had no history of urinary retention, fever, or abdominal pain. Physical examination was unremarkable. Blood work, including complete blood count (CBC), electrolytes, and urine culture, were all normal, except for low hemoglobin of 9.7 g/dL, as expected due to the CS and gross hematuria. The patient underwent computed tomography (CT) scan of the chest, abdomen, and pelvis with and without contrast, which revealed a large bladder mass measuring 6.1 x 6.0 x 6.0 cm, with no evidence of local or distant metastasis (Figures 1, 2).

**Figure 3:**
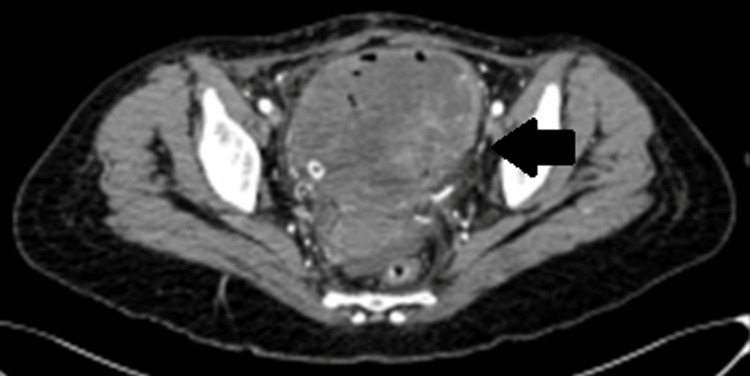
CT angiogram showing increase in the large irregular soft tissue density mass seen rising from the left lateral wall and base of the bladder, currently measuring 10 x 9.0 x 9.0 cm, while previously measuring 6.1 x 6.0 x 6.0 cm. CT, computed tomography

**Figure 4:**
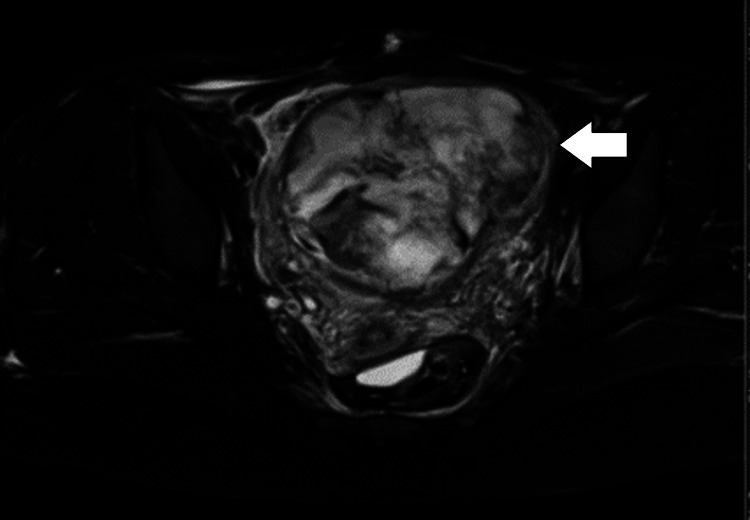
MRI of the pelvis showing previously noted large bladder tumor again seen arising from the left bladder wall. Normal enhancement of the bladder wall was seen with no convincing evidence to suggest an extramural invasion. Adjacent pelvic viscera appears to be displaced and invaded by this large bladder lesion. No large pelvic lymph nodes or destructive bony lesions were seen. MRI, magnetic resonance imaging

The patient subsequently underwent transurethral resection of the bladder tumor (TURBT) with the intention of obtaining pathological sample from the mass to diagnose the tumor. However, postoperatively, the patient had significant hematuria with clots requiring another TURBT to achieve adequate hemostasis, which was performed successfully. After a few days, the pathology reported the diagnosis of IMT; hence, multidisciplinary meeting was conducted, and it was concluded to perform partial cystectomy. Workup preoperatively included a CT angiogram and magnetic resonance imaging (MRI), which revealed an increase in tumor size (Figures 3, 4).

**Figure 5:**
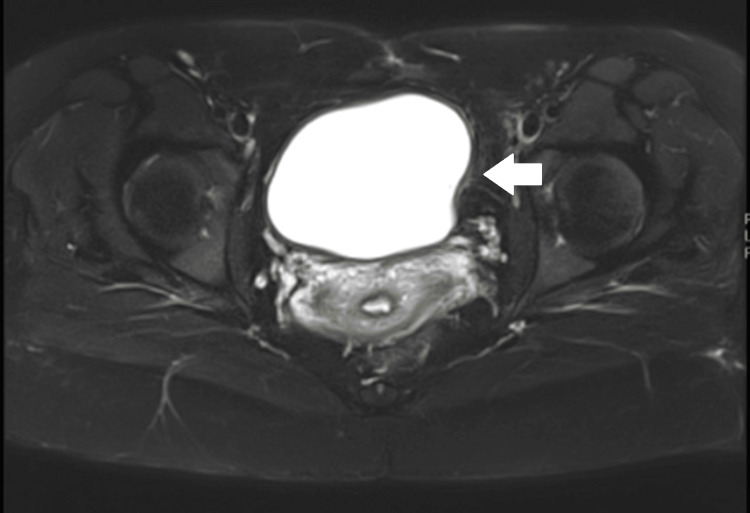
MRI of the pelvis. The urinary bladder is distended with persistent left lateral mild smooth wall thickening and adjacent surgical clips. No apparent abnormal diffusion restriction and suspicious findings are noted. MRI, magnetic resonance imaging

A partial cystectomy was performed five weeks after the admission, which was uneventful. During the operation, the tumor had a wide stalk, and the resection included the full thickness of the bladder wall with the perivesical fat surrounding it. In addition, the repair was performed in one layer using absorbable sutures. During the operation, the dissection was hard due to the recent gravid uterus. The patient subsequently had an uneventful postoperative course. Cystogram was performed two weeks postoperative, no leak or extravasation was noted, and Foley catheter was removed. The patient is being followed regularly in the clinic with regular radiological imaging for one year so far. She remains clinically well, and the last MRI performed fifteen months after the partial cystectomy revealed no recurrence or suspicious lesion in the bladder (Figure 5).

**Figure 6:**
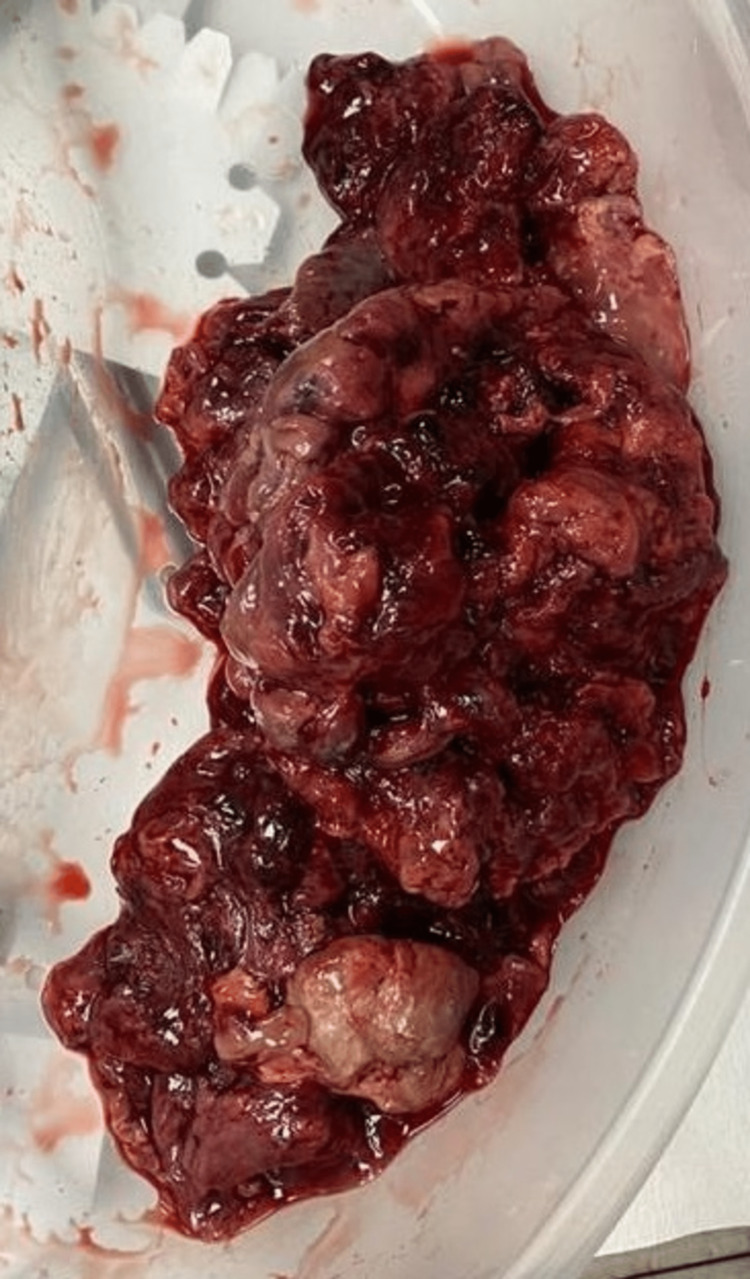
Grossly the tumor consists of multiple nodular pieces of gray-tan to brown hemorrhagic soft tissue measuring in aggregate 12.6 x 11 x 5.3 cm. The largest fragment measured 8.4 x 7.2 x 5.0 cm. Serial sectioning shows a white tan to the yellow trabeculated cut surface with grayish myxoid areas and hemorrhagic areas.

**Figure 7:**
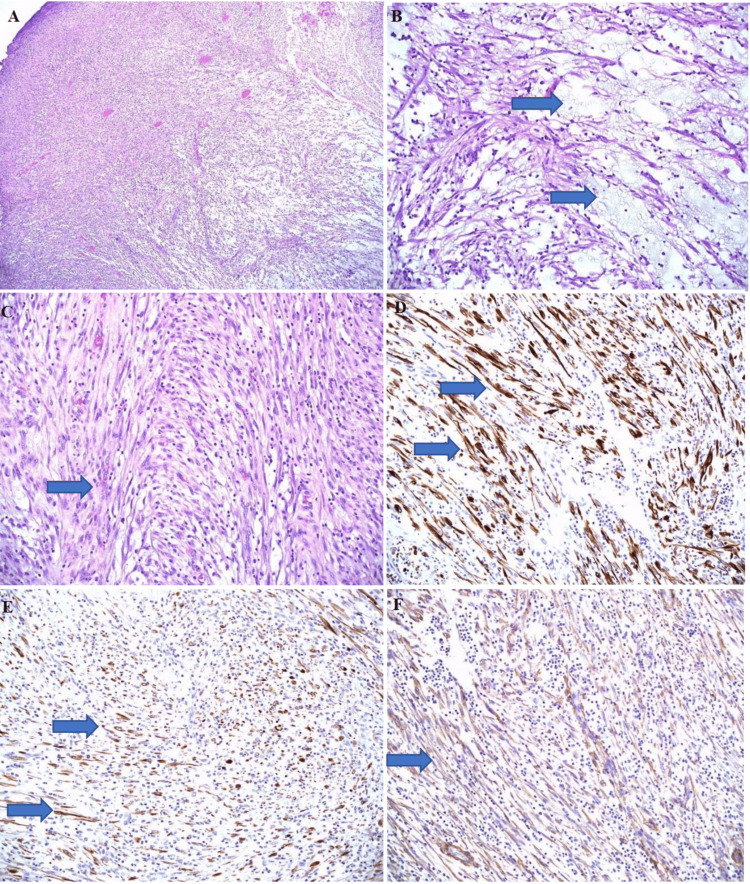
(A) Section of the bladder mass showing spindle myofibroblastic cell proliferation within the fibromyxoid matrix and edematous stroma. There are inflammatory cells in the background (hematoxylin and eosin, 200×). (B) Higher power showing bipolar cells within myxoid (arrow) and edematous stroma (hematoxylin and eosin, 400×). (C) Higher power showing spindle cells (arrow) with eosinophilic cytoplasm, elongated nuclei with smooth contours, and open chromatin (hematoxylin and eosin, 400×). (D) Focal staining (arrow) for desmin (immunohistochemistry stain, 400×). (E) Focal staining (arrow) for pankeratin (immunohistochemistry stain, 400×). (F) Focal staining (arrow) for H-caldesmon (Immunohistochemistry stain, 400×).

Macroscopic examination showed the tumor consisting of multiple nodular pieces of gray-tan to brown hemorrhagic soft tissue measuring in aggregate 12.6 x 11 x 5.3 cm (Figure 6). The pathological diagnosis revealed an IMT, with negative margins. Microscopic examination revealed a tumor composed of spindle myofibroblastic cell proliferation within the fibromyxoid matrix and edematous stroma. There were mixed inflammatory cells in the background composed of lymphocytes, plasma cells, and eosinophils. In some areas, spindle cells showed a compact fascicular pattern with eosinophilic cytoplasm, elongated nuclei with smooth contours, and open chromatin. The immunohistochemistry test was positive for vimentin, pan-cytokeratin (Pan-CK), CAM5.2, desmin, smooth muscle actin (SMA), and muscle-specific actin (MSA). Negative staining was obtained with anaplastic lymphoma kinase (ALK), P63, ALK-1, CD34, Myod1, HMB45, and MART1. Myogenin and Myo-D satins were also negative, which was helpful in differentiating the lesion from rhabdomyosarcoma. The focal staining for desmin, SMA, and MSA, and the negative staining for P63 were helpful n differentiating this tumor from sarcomatoid carcinoma. Immunohistochemistry stains showed overlapping features with smooth muscle neoplasm. However, the overall morphological features were more in favor of IMT (Figure 7). The fluorescence in situ hybridization (FISH) study showed no rearrangement of the ALK gene region. Lymph nodes were negative for metastasis.

## Discussion

IMT is a rare intermediate tumor composed of myofibroblastic spindle cells with an inflammatory infiltrate of plasma cells, lymphocytes, and eosinophils [1]. IMT and other tumors of this nature were described as having an unknown neoplastic potential. Brunn first described these kinds of tumors in 1937 from a sample of a lung, which remains the most common anatomical site for these kinds of tumors until now [3]. IMT is extremely rare to develop in the genitourinary tract and was first described in 1980 by Joel and Roth [4].

Until 2014, only 182 cases of IMT were reported in the urinary bladder, which were collected in the systematic review of Teoh et al. In terms of manifestations, the most common presentation among those 182 patients was hematuria, which occurred in approximately 72% of the cases, followed by dysuria (19.8%), increased frequency (18.8%), abdominal pain (13.5%), and flank pain (2.1%). Of the total number of patients, 3.1% initially presented with hemodynamic instability. No patient developed hydronephrosis or had abnormal urine cytology. In addition, 6.3% of the patients had anemia, and 1.1% developed abnormal renal function. The most common tumor site among these patients was the posterior wall of the bladder (33.3%), followed by the dome of the bladder (22.2%), right lateral wall (19.4%), left lateral wall (13.9%), anterior wall (11.1%), and was never reported in the trigone. The mean size of the tumors in all 182 cases was 4.48 ± 2.12 cm [5]. In our case, the patient presented with severe hematuria, and the tumor was found in the left lateral wall.

There are no known predisposing factors to develop IMT in the bladder. However, some papers found associations related to autoimmune diseases, infections, traumatic injuries, and recent history of surgical instrumentation [6]. IMT cases were reported in both genders, ranging from infancy until 88 years [6]. However, IMT occurs in young persons and is more slightly to happen in males, with a male-to-female ratio of 4:3 [7].

IMT might mimic some malignancies in morphology and immunology; hence, diagnosis might be challenging for pathologists. In addition, the expression of immunohistochemistry of myogenic and epithelial markers might be present, and these markers are present in several cancers, such as leiomyosarcoma and rhabdomyosarcoma, which might mislead the pathologist. However, the use of ALK by immunohistochemistry might be helpful in the diagnosis, as it is positive and overexpressed in IMT in 40-60% of the cases [8]. Moreover, detecting ALK gene translocation using FISH may help in diagnosis, as it is present in almost 50% of IMT cases [9]. In our case, the tumor was negative for ALK and FISH. However, the diagnosis of IMT was favored based on the overall morphological features.

The standard treatment offered for most patients is TURBT. In the systematic review of Teoh et al., 60.8% of the patients underwent TURBT, followed by partial cystectomy in 29.2%, then radical cystectomy in 9.2%, and eventually biopsy through cystoscopy in 0.8%. Of those who underwent TURBT, 5.5% underwent another TURBT, 24.7% underwent partial cystectomy, and 1.4% underwent radical cystectomy [5]. Our patient was first managed with TURBT with the intention of obtaining a pathological sample from the mass, but due to the constant bleeding from the tumor, she underwent another TURBT for hemostasis purpose. After the result of the pathology, a multidisciplinary meeting was conducted, and it was concluded to perform a partial cystectomy.

Nonresectable IMT might be treated pharmacologically since cyclooxygenase 2 (COX2), vascular endothelial growth factor (VEGF), and ALK that IMTs express may be targeted by medications [10]. For COX2, the use of nonsteroidal anti-inflammatory drugs was reported in the literature in certain situations [11]. ALK expression also might be targeted by crizotinib [12]. In our case, the tumor was surgically resectable, and therefore we did not investigate the possibility of pharmacotherapy.

## Conclusions

IMT is a rare tumor in the genitourinary tract that is diagnosed by tissue biopsy. The most common presentation is hematuria, followed by dysuria. IMT has no known predisposing factors, and the age distribution of the disease ranges from infancy until old age. IMT has many mimickers, for which a highly skilled pathologist is needed for an accurate diagnosis. Treatment is mainly surgical in the form of TURBT or partial cystectomy in large masses, while pharmacological options are still valid for some instances. Our patient was treated by a partial cystectomy, and an MRI performed 15 months after operation revealed no recurrence.

## References

[1] Coffin C, Fletcher JA (2002). Inflammatory myofibroblastic tumour. Pathology and Genetics of Tumours of Soft Tissue and Bone, 4th Edition, Volume 5.

[2] Patnana M, Sevrukov AB, Elsayes KM, Viswanathan C, Lubner M, Menias CO (2012). Inflammatory pseudotumor: the great mimicker. AJR Am J Roentgenol.

[3] Brunn H (1939). Two interesting benign lung tumors of contradictory histopathology: remarks on the necessity for maintaining the chest tumor registry. J Thorac Surg.

[4] Joel A, Roth M (1980). Reactive pseudosarcomatous response in urinary bladder. Urology.

[5] Teoh JY, Chan NH, Cheung HY, Hou SS, Ng CF (2014). Inflammatory myofibroblastic tumors of the urinary bladder: a systematic review. Urology.

[6] Gleason BC, Hornick JL (2008). Inflammatory myofibroblastic tumours: where are we now?. J Clin Pathol.

[7] Yagnik V, Chadha A, Chaudhari S, Patel K (2010). Inflammatory myofibroblastic tumor of the urinary bladder. Urol Ann.

[8] Cessna MH, Zhou H, Sanger WG (2002). Expression of ALK1 and p80 in inflammatory myofibroblastic tumor and its mesenchymal mimics: a study of 135 cases. Mod Pathol.

[9] Coffin CM, Patel A, Perkins S, Elenitoba-Johnson KS, Perlman E, Griffin CA (2001). ALK1 and p80 expression and chromosomal rearrangements involving 2p23 in inflammatory myofibroblastic tumor. Mod Pathol.

[10] Applebaum H, Kieran MW, Cripe TP (2005). The rationale for nonsteroidal anti-inflammatory drug therapy for inflammatory myofibroblastic tumors: a Children's Oncology Group study. J Pediatr Surg.

[11] Chavez C, Hoffman MA (2013). Complete remission of ALK-negative plasma cell granuloma (inflammatory myofibroblastic tumor) of the lung induced by celecoxib: a case report and review of the literature. Oncol Lett.

[12] Butrynski JE, D'Adamo DR, Hornick JL (2010). Crizotinib in ALK-rearranged inflammatory myofibroblastic tumor. N Engl J Med.

